# Dietary Supplementation with *Deinococcus radiodurans* Extract Alleviates Obesity and Systemic Inflammation via Gut Microbiota Modulation in Murine and Feline Models

**DOI:** 10.3390/ani16132072

**Published:** 2026-07-05

**Authors:** Wangyang Hu, Yan Wang, Cong Hua, Chenxiang Shi, Yifei Tu, Shaotang Ye, Min Hu, Qiang Huang, Lin Lin, Yuejin Hua

**Affiliations:** 1 MOE Key Laboratory of Biosystems Homeostasis and Protection, Institute of Biophysics, College of Life Sciences, Zhejiang University, Hangzhou 310058, China; huwangyang@mhdx.cn (W.H.); 11916015@zju.edu.cn (C.S.); 2 MetaHealth Technology (Hangzhou) Co., Ltd., Hangzhou 310016, China; wangyan@mhdx.cn (Y.W.); huacong@mhdx.cn (C.H.); tuyifei@mhdx.cn (Y.T.); yeshaotang@mhdx.cn (S.Y.); humin@mhdx.cn (M.H.); 3 Jiangxi Zhenghe Environmental Protection Engineering Co., Ltd., Nanchang 330001, China; qiangh5@126.com

**Keywords:** *Deinococcus radiodurans*, obesity, SCFA, *Oscillibacter*, gut microbiota, metabolic health, weight management

## Abstract

This study investigated the metabolic regulatory effects and underlying microbial mechanisms of *Deinococcus radiodurans* extract (DRE), using high-fat diet (HFD)-induced obese mice as the primary mechanistic model and naturally overweight felines. In the mouse model, a 1.5% DRE supplementation mitigated HFD-induced obesity, reduced serum total cholesterol and low-density lipoprotein levels, and markedly ameliorated hepatic steatosis. Fecal 16S rRNA gene sequencing revealed that DRE effectively reversed murine microbial dysbiosis by significantly restoring core commensals depleted by the HFD, notably *Ureaplasma* and the short-chain fatty acid (SCFA)-producer *Odoribacter*, while concurrently suppressing the overgrowth of obesity-associated taxa including *Alloprevotella* and *Phascolarctobacterium*. As a translational complement, a 28-day DRE intervention in felines under isocaloric maintenance conditions, with no significant change in body weight, body condition score, or fecal score, validated these systemic benefits, significantly enhancing serum total antioxidant capacity by 16.1% and reducing the systemic inflammatory marker serum amyloid A by 27.8%, indicating that the antioxidant and anti-inflammatory effects of DRE are independent of weight change. Concurrently, feline fecal microbiota profiling demonstrated a parallel ecological remodeling, characterized by the enrichment of potent SCFA producers (*Oscillibacter* and the *[Eubacterium]_hallii_group*) and the profound suppression of the pro-inflammatory pathogen *Fusobacterium*. Collectively, by integrating deep mechanistic insights from mice with translational evidence from felines, this study demonstrates that DRE exerts comprehensive anti-obesity and anti-inflammatory effects by regulating lipid metabolism and reshaping the gut microbiota, establishing its robust potential as a novel functional ingredient for metabolic health in companion animals.

## Simple Summary

Obesity is a significant metabolic burden affecting both companion animals and humans. This study focused on the metabolic regulatory effects of a natural extract derived from *Deinococcus radiodurans* (DRE). Using a high-fat diet-induced obese mouse model as the core, we conducted an in-depth investigation into the molecular and metabolic mechanisms by which DRE mitigates weight gain, alleviates hepatic steatosis, improves lipid profiles, and reshapes gut microbiota. Complementing this, a translational study in naturally overweight domestic cats (BCS > 6/9) was performed to validate the findings, demonstrating that DRE intervention under stable energy intake significantly enhances antioxidant capacity and reduces systemic inflammation. Collectively, by integrating mechanistic insights from mice with translational evidence from cats, this study highlights DRE as a safe and potent functional ingredient, offering a promising nutritional strategy for the pet health industry to combat obesity-related metabolic disorders.

## 1. Introduction

The number of overweight and obese individuals has increased significantly across the globe in recent years [1]. As a chronic metabolic condition, obesity can lead to serious health issues [2]. The primary cause is often a long-term imbalance where energy intake from food is higher than the energy the body consumes, leading to fat deposition [3,4,5]. Obesity is typically characterized by weight gain, elevated lipid levels, and increased oxidative stress [6]. These factors represent a threat to the long-term health of both humans and companion animals [7,8,9], exacerbating the risk of metabolic comorbidities such as insulin resistance and orthopedic disorders in canine and feline populations [10,11,12].

Current methods for managing weight primarily include changes in lifestyle, the use of certain drugs, or surgery [13,14,15]. While reducing the intake of high-fat foods and increasing exercise can be effective, they are often difficult to maintain. Some drugs are available to reduce nutrient absorption or suppress appetite, but they may cause side effects such as diarrhea or endocrine issues [16,17,18]. Surgical options are expensive and can lead to complications such as inflammation or imbalances in the gut microbiota [19]. Therefore, using safe and effective dietary supplements is a practical way to manage obesity with fewer side effects, as natural bioactive extracts have shown considerable promise in modulating lipid metabolism without adverse pharmacological burdens [20,21,22,23].

The intestinal microbiota is a complex ecosystem that helps regulate the energy balance of the host [24,25,26]. A healthy gut community is important for maintaining overall health [27,28]. Imbalances in these microorganisms can lead to energy disorders and contribute to the development of obesity [29]. Studies have shown that a high-fat diet can reduce microbial diversity and change the structure of the gut community [30,31,32,33]. Conversely, weight management and healthy diets are often linked to an increase in beneficial bacteria and improved species richness [34,35].

*Deinococcus radiodurans* is a unique extremophilic bacterium distinguished by its extraordinary resilience to ionizing radiation and oxidative stress, traits primarily attributed to its highly efficient DNA repair mechanisms and robust antioxidant systems [36,37,38,39], including the production of potent carotenoids like deinoxanthin that actively scavenge reactive oxygen species [40]. Among these protective systems, a distinctive feature of D. radiodurans is its accumulation of small-molecule antioxidant complexes—including the C40 carotenoid deinoxanthin, Mn^2+^-organic ligand (peptide and phosphate complexes), and stress-response proteins such as PprI—which collectively render this bacterium exceptionally resistant to oxidative insults [41,42]. We hypothesized that the fermentation-derived DRE preserves these bioactive compounds within a protective matrix that may partially shield them from upper-GI degradation, enabling delivery to the distal gut where they could modulate the resident microbiota and improve metabolic health. To test this hypothesis, we examined the effects of DRE supplementation on obesity, systemic inflammation, and gut microbial ecology in both mice and cats.

Consequently, the regulatory effects of DRE on weight management, lipid profiles, and the gut microenvironment were investigated using HFD-induced obese mouse and feline models. Through the analysis of growth performance, histopathological shifts in the liver and adipose tissues, and the metabolic output of the gut microbiota, this research provides the theoretical and experimental evidence necessary for the development of *D. radiodurans* derivatives as functional ingredients. These findings serve as a foundation for utilizing special microbial resources in the formulation of bioactive products targeted at modulating metabolic health and mitigating obesity-related conditions, offering a novel postbiotic strategy for translational application in both laboratory models and companion animal nutrition [43].

## 2. Materials and Methods

### 2.1. Culture and Preparation of D. radiodurans

*Deinococcus radiodurans* (DR) was activated on Tryptone Glucose Yeast (TGY) agar at 28 °C for 36 h. A single colony was then transferred to TGY broth and cultured at 30 °C (220 rpm) until reaching the log phase. This liquid culture served as the seed for solid-state fermentation. The solid substrate, consisting of 60% soybean meal and 40% sunflower meal, was sterilized and cooled before inoculation with the seed culture at a 10% (*v*/*w*) ratio.

Fermentation was carried out at 28 °C for 3–5 days. The resulting product was dried, ground through an 80-mesh sieve, and extracted using 75% ethanol. After concentration and freeze-drying, the final DRE powder was obtained. The moisture content of the final product was maintained below 10%. DRE is characterized as a postbiotic, as it comprises a non-viable, processed fermentation product derived from *D. radiodurans*.

### 2.2. Mice Experimental Design and Sample Collection

Thirty-two C57BL/6J mice (7–8 weeks old, 20 ± 1 g) were acclimated for one week and maintained under a 12 h light/dark cycle at 23–25 °C and 55 ± 5% humidity with free access to food and water. The mice were assigned to four groups: control (CON, *n* = 6), high-fat diet (HFD, *n* = 10), HFD supplemented with 0.5% *D. radiodurans* extract (DRE, *n* = 8), and HFD supplemented with 1.5% DRE (*n* = 8). The experimental period lasted 20 weeks, consisting of a 12-week obesity induction phase followed by an 8-week intervention phase where the DRE groups received their respective treatments alongside the HFD. The DRE doses of 0.5% and 1.5% (*w*/*w*) were selected based on the typical inclusion range (0.1–2.0%) reported for postbiotic and fermentation-derived extracts in HFD-fed murine models [44], together with safety data from previous studies using *D. radiodurans*-derived preparations [45,46]. Based on a standard daily feed intake of approximately 3 g per mouse, these dietary concentrations correspond to approximately 15 mg/day (0.5%) and 45 mg/day (1.5%) per mouse, bracketing a low-dose and high-dose range to evaluate potential dose-dependent efficacy. No signs of toxicity or reduced food intake were observed at either dose throughout the 20-week intervention.

At weeks 12, 16, and 20, mice were deeply anesthetized via intraperitoneal injection of pentobarbital sodium. Once deep anesthesia was confirmed, blood samples were collected via the retro-orbital sinus. A portion of whole blood was collected in EDTA tubes for routine blood analysis. The remaining blood was centrifuged at 3000 *g* for 10 min at 4 °C to separate serum for the measurement of total serum cholesterol (TC), triglycerides (TG), and low-density lipoprotein (LDL-C), serum concentrations of TC, TG, and LDL-C were quantified using corresponding mouse-specific commercial enzyme-linked immunosorbent assay (ELISA) kits (Cat. No. ml092733 for TC, ml092640 for LDL-C, and ml076637 for TG; Shanghai Enzyme-linked Biotechnology Co., Ltd., Shanghai, China) in strict accordance with the manufacturers’ instructions. Immediately following blood collection, all mice were humanely euthanized by cervical dislocation to ensure a painless death prior to tissue harvesting. Subsequently, liver and inguinal white adipose tissue (WAT) were excised, weighed, rinsed in ice-cold phosphate-buffered saline (PBS), and immediately fixed for pathological observation and Oil Red O staining.

Histopathological evaluations were conducted to assess hepatic steatosis and adipose tissue morphology in mice. Tissue samples were collected at both the 16-week and 20-week time points during the experimental period. Following euthanasia, liver and inguinal white adipose tissue (WAT) were excised, rinsed in ice-cold phosphate-buffered saline (PBS), and immediately fixed.

Fresh fecal samples were collected at week 20 immediately snap-frozen, and stored at −80 °C for subsequent intestinal microbiota analysis. To minimize inter-individual biological variation and ensure sufficient biomass for subsequent microbial DNA extraction, a 2-by-2 pooling strategy was employed within each group. Specifically, fecal samples from every two mice in the same group were thoroughly mixed and pooled into a single composite sample. Consequently, a total of 12 independent composite samples were subjected to 16S rRNA gene sequencing, including the control group (CON, *n* = 3 composite samples, derived from 6 mice), the high-fat diet group (HFD, *n* = 5 composite samples, derived from 10 mice), and the DRE intervention group (HFD + 1.5% DRE, *n* = 4 composite samples, derived from 8 mice). All composite samples were immediately frozen in liquid nitrogen upon collection and stored at −80 °C until further processing and DNA extraction.

Tissue Fixation and Sectioning: Fresh tissue samples were fixed in 4% paraformaldehyde fixative solution for 24 h at room temperature to preserve tissue architecture. After fixation, tissues were dehydrated through a graded ethanol series, cleared in xylene, and embedded in paraffin wax. Embedded tissues were sectioned at a thickness of 5 μm using a microtome and mounted onto glass slides for hematoxylin and eosin (H&E) staining.

H&E Staining Procedure: Paraffin-embedded sections were deparaffinized in xylene and rehydrated through a graded ethanol series (100%, 95%, 80%, and 70%) to distilled water. Sections were then stained with Mayer’s hematoxylin solution for 5–8 min to visualize nuclei, followed by a 10-min rinse in running tap water for bluing. Subsequently, sections were counterstained with eosin solution for 2–3 min to stain the cytoplasm and extracellular matrix. After staining, sections were dehydrated through graded ethanol, cleared in xylene, and mounted with a resinous mounting medium. Images were captured using a light microscope (Olympus Corporation, Tokyo, Japan) equipped with a digital camera. Adipocyte size and morphology were assessed, and at least five fields per sample were analyzed.

Oil Red O Staining Procedure: For the assessment of neutral lipid accumulation in the liver, fresh liver tissue samples were embedded in optimal cutting temperature (OCT) compound and frozen at −80 °C. Frozen sections were cut at a thickness of 8–10 μm using a cryostat and air-dried for 10–15 min. Sections were fixed in 4% paraformaldehyde fixative for 15 min at room temperature, rinsed with distilled water, and then washed with 60% isopropanol. The fixed sections were stained with freshly prepared Oil Red O working solution for 10–15 min at room temperature. Oil Red O stock solution was prepared by dissolving 150 mg of Oil Red O powder in 50 mL of 100% isopropanol, heated to dissolve completely, and filtered to obtain a 3 mg/mL stock solution. The working solution was prepared by mixing 15 mL of stock solution with 10 mL of distilled water, followed by filtration. After staining, sections were differentiated in 60% isopropanol to remove background staining, rinsed briefly in distilled water, and counterstained with Mayer’s hematoxylin for 1–2 min to visualize nuclei. Sections were then washed in tap water and mounted in an aqueous mounting medium. Images were captured using a light microscope. The area of Oil Red O-positive lipid droplets was quantified using ImageJ software (ImageJ software (version 1.54g, National Institutes of Health, Bethesda, MD, USA)) and expressed as a percentage of the total field area. At least five random fields per sample were analyzed.

The control diet (FB-D12450J, 10% fat by energy, 3850 kcal/kg) and high-fat diet (FB-D12492, 60% fat by energy, 5240 kcal/kg) were custom-formulated by Wuxi Fanbo Biotechnology Co., Ltd. (Wuxi, China). The DRE-supplemented diets were prepared by adding DRE powder at 0.5% (*w*/*w*) or 1.5% (*w*/*w*) to the complete FB-D12492 formulation, with all other ingredients proportionally reduced to maintain a 100% (*w*/*w*) total. Complete ingredient lists and proximate compositions are provided in Supplementary Table S3.

### 2.3. Feline Experimental Design and Sampling

A total of six clinically healthy, overweight adult cats (Body Condition Score [BCS] > 6/9) with no prior history of systemic disease or metabolic disorders were enrolled in this study to evaluate the metabolic and physiological impacts of the nutritional intervention, cats were purchased from several catteries in Hangzhou, China. The cohort consisted of five British Shorthairs and one Bengal cat of similar age and baseline body weight (aged 4–5 years; 3 spayed females, 3 neutered males; detailed individual characteristics are provided in Supplementary Table S1). Prior to the trial, all cats were confirmed to be clinically healthy via comprehensive veterinary examinations and routine serum xqbiochemistry, ensuring no history of metabolic disorders or recent pharmacological treatments within the preceding 6 months.

To minimize environmental, behavioral, and physical activity confounding factors, all subjects were individually housed in standard enclosures within a single climate-controlled facility. Environmental parameters were maintained strictly at a temperature of 22 ± 2 °C, a relative humidity of 50 ± 10%, and a 12 h:12 h light–dark cycle. This standardized housing protocol strictly monitored dietary intake while uniformly restricting spontaneous physical activity, thereby ensuring a consistent sedentary baseline across the cohort throughout the entire study duration.

To strictly eliminate dietary and environmental confounding factors, a 4-week nutritional acclimation phase was implemented prior to the experimental intervention. Throughout the entire study (including both the 4-week acclimation and the 28-day intervention phases), the cats were fed exclusively a standardized commercial extruded diet (HALO Adult Chicken Formula; Guaranteed analysis: ≥32% crude protein, ≥16% crude fat, crude fiber ≤ 5%, moisture ≤ 10%, crude ash ≤ 7%, taurine ≥ 0.1%, and 3780 kcal/kg metabolizable energy). The daily caloric intake for each cat was individually calculated in accordance with the 2021 AAHA/AAFP Feline Life Stage Guidelines to meet their specific maintenance energy requirements.

Crucially, the daily caloric intake for each cat was individually calculated based on their specific maintenance energy requirements and precisely weighed (ranging from 74 g to 80 g/day) to ensure that the isocaloric intake remained strictly constant before and during the trial. This experimental design guarantees that physiological, oxidative, or microbial alterations observed are completely independent of caloric restriction or changes in basal diet composition.

Following the acclimation phase, the 28-day intervention phase commenced. During this period, each cat was fed twice daily at 08:00 and 18:00. At 08:00, 0.75 g (1.5%) of D. radiodurans extract (DRE) powder was thoroughly mixed with a small portion of crushed kibble and administered to ensure complete ingestion, followed immediately by the remainder of the morning ration. At 18:00, the cats were provided with their scheduled daily ration without supplementation. Fresh water was provided ad libitum throughout the study.

Body weight, waist circumference, and BCS were recorded weekly. These clinical metrics were used to thoroughly assess the safety and overall physical status of the cats during the intervention. On days 0 and 28 of the trial, blood samples were collected from each cat via cephalic venipuncture into standard serum separator tubes by licensed veterinary personnel. The blood samples were centrifuged at 3500 *g* for 15 min at room temperature to isolate serum, which was then aliquoted and stored at −80 °C for downstream biochemical and molecular analyses. Concurrently, fresh fecal samples were collected at the start (day 0) and the end (day 28) of the trial, immediately snap-frozen, and stored at −80 °C for subsequent intestinal microbiota analysis. Each individual cat’s fecal sample at each timepoint was subjected to 16S rRNA gene sequencing without pooling or compositing, yielding *n* = 6 paired samples per timepoint (i.e., 6 samples at D0 and 6 samples at D28). Paired pre/post microbiota changes within individual animals were tested using the Wilcoxon signed-rank test.

To evaluate the alterations in systemic redox status under the isocaloric regimen, feline serum levels of key antioxidant enzymes and lipid peroxidation products were determined. Specifically, the activities of superoxide dismutase (SOD) and catalase (CAT), alongside total antioxidant capacity (T-AOC) and malondialdehyde (MDA) concentrations, were quantified using specific commercial assay kits (Cat. No. A001-3-2 for SOD, A007-1-1 for CAT, A015-2-1 for T-AOC, and A003-1-2 for MDA; Nanjing Jiancheng Bioengineering Institute, Nanjing, China) according to the manufacturer’s protocols.

Additionally, the systemic inflammatory response was meticulously assessed. The feline serum concentrations of serum amyloid A (SAA), interleukin-6 (IL-6), interferon-gamma (IFN-γ), tumor necrosis factor-alpha (TNF-α), and interleukin-10 (IL-10) were quantified using corresponding feline-specific commercial enzyme-linked immunosorbent assay (ELISA) kits (Cat. No. ml109133 for SAA, ml023093 for IL-6, ml023105 for IFN-γ, ml031931 for TNF-α, and ml023095 for IL-10; Shanghai Enzyme-linked Biotechnology Co., Ltd., Shanghai, China) in strict accordance with the manufacturers’ instructions.

### 2.4. Animal Ethics and Welfare

All animal experiments, including both murine and feline studies, were conducted in strict accordance with the guidelines for the care and use of laboratory animals and were formally approved by the Animal Ethics Committee of Zhejiang A&F University (Approval No. ZAFUAC2025010). All efforts were made to minimize animal suffering and to optimize the number of animals used in the study.

### 2.5. Gut Microbial Diversity Analysis

Total genomic DNA was extracted from the fecal samples using the GHFDE100 DNA isolation kit (GUIHE Laboratories, Hangzhou, China) in accordance with the manufacturer’s instructions. Extracted DNA was stored at −20 °C until further analysis. The quantity and quality of the extracted DNA were determined using a NanoDrop ND-1000 spectrophotometer (Thermo Fisher Scientific, Waltham, MA, USA) and agarose gel electrophoresis, respectively.

The hypervariable V4 region of the bacterial 16S rRNA gene was amplified using the specific primers 515F (5′-GTGCCAGCMGCCGCGGTAA-3′) and 806R (5′-GGACTACHVGGGTWTCTAAT-3′). Sample-specific paired-end 6-bp barcodes were incorporated into the TrueSeq adaptors for multiplex sequencing. The PCR reactions were performed in a total volume containing 25 μL Phusion High-Fidelity PCR Master Mix (2×), 3 μL (10 μM) of each primer, 10 μL DNA template, 3 μL DMSO, and 6 μL ddH_2_O. Thermal cycling conditions consisted of an initial denaturation at 98 °C for 30 s, followed by 25 cycles of denaturation at 98 °C for 15 s, annealing at 58 °C for 15 s, and extension at 72 °C for 15 s, with a final extension at 72 °C for 1 min. Negative controls (no template) were included in each PCR run. PCR amplicons were purified using Agencourt AMPure XP Beads (Beckman Coulter, Indianapolis, IN, USA) and quantified using the PicoGreen dsDNA Assay Kit (Invitrogen, Carlsbad, CA, USA). After the individual quantification step, amplicons were pooled in equal amounts, and paired-end sequencing (2 × 150 bp) was performed on the DNBSEQ-G99 platform (MGI Tech Co., Ltd., Shenzhen, China).

Quality filtering of raw paired-end reads was conducted using Vsearch (v2.22.1) under predefined filtering criteria to retain high-quality clean reads. The filtered reads were subsequently aligned to the SILVA reference database with the UCHIME algorithm for chimera detection and elimination, yielding the final effective tags. Amplicon sequence variants (ASVs) were generated from effective tags using the UNOISE2 denoising algorithm. To reduce noise from low-abundance taxa, ASVs with a mean relative abundance < 0.01% across all samples were removed prior to downstream analysis.

The alpha-diversity indices (Chao1 richness and Shannon diversity) were calculated using QIIME2 (v2024.10) from the ASV table normalized via the total sum scaling (TSS) method and standardized to a total abundance of 100,000 reads per sample. Group-level differences in alpha-diversity were tested via the Kruskal–Wallis test, followed by pairwise comparisons with FDR correction. For beta-diversity, weighted UniFrac distances and Bray–Curtis dissimilarities were calculated and visualized via Principal Coordinate Analysis (PCoA). The statistical significance of microbial community separation among groups was assessed by Permutational Multivariate Analysis of Variance (PERMANOVA) using the R package ‘vegan’ (v2.6, R v3.6.3) [47]. Differential abundance analysis of bacterial taxa (at the genus level) was performed using ANCOM-BC2 (Analysis of Compositions of Microbiomes with Bias Correction), which accounts for phylogenetic relationships and batch effects. For the feline study, paired differences in specific genera between Day 0 and Day 28 were further tested using the Wilcoxon signed-rank test (non-parametric paired comparison). Taxa with adjusted *p*-value < 0.05 (FDR-corrected) were considered significantly differentially abundant.

### 2.6. Statistical Analysis

Data Reporting and Overall Significance Thresholds: Data are reported as mean ± standard deviation (SD) unless otherwise noted. All statistical analyses were performed using SPSS version 20.0. A *p*-value less than 0.05 was considered statistically significant, and exact *p*-values are reported to three decimal places where available, with *p* < 0.001 denoted when below this threshold.

Single-Timepoint, Multi-Group Comparisons (Cross-Sectional Data): For single-timepoint measurements across multiple groups (e.g., serum lipid profiles [total cholesterol, triglycerides, LDL cholesterol] at weeks 12, 16, and 20; liver weight; Oil Red O quantification; adipocyte morphometry; and serum antioxidant/inflammatory markers in mice), one-way ANOVA was performed to test for overall group differences, followed by Tukey’s post-hoc test for pairwise comparisons.

**Figure 1:**
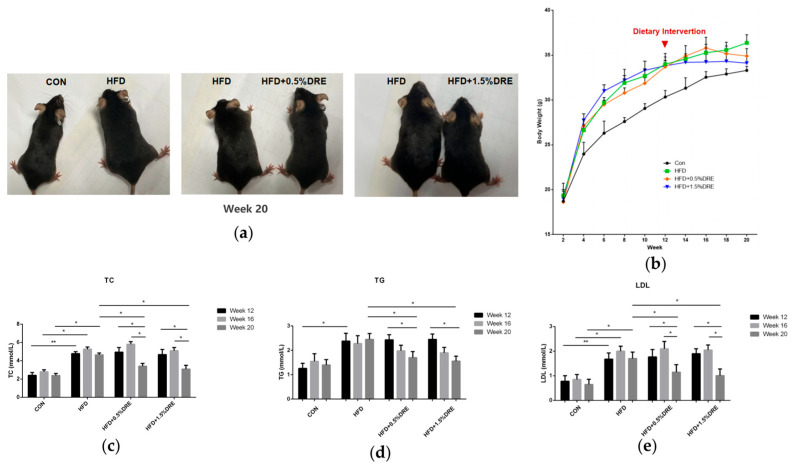
Effects of DRE on the growth performance and hematological parameters of mice. (**a**) Physical appearance of mice from different groups. (**b**) Body weight changes over 20 weeks (*n* = 10 per group). The red triangle indicates the initiation of dietary intervention at week 12. (**c**) Serum total cholesterol (TC) concentration at weeks 12, 16, and 20 (*n* = 6 per group). (**d**) Serum triglyceride (TG) concentration at weeks 12, 16, and 20 (*n* = 6 per group). (**e**) Serum low-density lipoprotein (LDL) concentration at weeks 12, 16, and 20 (*n* = 6 per group). All data are expressed as the mean ± SD. The horizontal lines with asterisks indicate statistical significance between the connected groups (* *p* < 0.05, ** *p* < 0.01).

Longitudinal Body-Weight Measurements in Mice (Repeated Measures): For body-weight measurements tracked longitudinally across weeks 0–20 (Figure 1b), a two-way repeated-measures ANOVA was employed with group (CON, HFD, HFD + 0.5% DRE, HFD + 1.5% DRE) and time (weeks 0, 4, 8, 12, 16, 20) as between- and within-subject factors, respectively. Tukey’s post-hoc test was applied to pairwise group × time interactions to identify specific timepoints where groups diverged significantly.

Feline Pre/Post Paired Comparisons: For the feline pre/post intervention design (Day 0 vs. Day 28), paired measurements were analyzed using appropriate tests based on data distribution and variable type. Normally distributed continuous variables (body weight, serum antioxidant capacity [T-AOC], and inflammatory markers [serum amyloid A, TNF-α, IL-6, IFN-γ, IL-10]) were compared using the paired Student’s *t*-test. For non-normally distributed data and ordinal variables (body condition score [BCS] and fecal score), the non-parametric Wilcoxon signed-rank test was utilized.

Microbiota Statistical Methods: Statistical testing of microbiota alpha-diversity and compositional shifts, including Kruskal–Wallis and Dunn post-hoc tests (with FDR correction), PERMANOVA, and ANCOM-BC2 differential abundance analysis, is described in detail in Section 2.5 (Gut Microbiota Analysis).

## 3. Results

### 3.1. Effect of DRE on Growth Performance, Body Weight, and Hematological Parameters in Mice

To evaluate the influence of DRE on the physiological status of obese mice, morphological changes, body weight, and serum lipid parameters were monitored (Figure 1). Mice in the HFD group displayed a noticeably larger body size compared to the CON group after 20 weeks of feeding (Figure 1a). At the 12th week, the body weight of the HFD group was 10.1% higher than that of the CON group, confirming the successful establishment of the high-fat-induced obesity model from a morphological perspective (Figure 1b). Following the initiation of dietary intervention at week 12, DRE treatment mitigated further weight gain in a dose-dependent manner. By the end of the 20th week, the average body weight of mice in the 0.5% DRE group was 1.47 g (4.0%) lower than that of the HFD group. In contrast, the 1.5% DRE group showed a more pronounced reduction in weight gain, with an average weight 2.25 g (6.2%) lower than the HFD group, suggesting that higher concentrations of DRE are more effective in weight management.

The serum lipid analysis further supported the protective role of DRE against metabolic disturbances induced by HFD. Crucially, prior to the dietary intervention at week 12, high-fat intake had already led to significantly elevated levels of total cholesterol (TC) and low-density lipoprotein (LDL) in the HFD group compared to the CON group, which was highly consistent with the characteristics of HFD-induced metabolic syndrome (Figure 1c,e). Following the intervention, DRE significantly enhanced the reduction of TC levels. Both DRE-treated groups exhibited decreased TC concentrations, with the 1.5% dose demonstrating superior efficacy, indicating a potential dose-dependent response (Figure 1c). As for TG levels, the 1.5% DRE treatment improved the levels at the end of the experiment, while the 0.5% DRE treatment showed no obvious effect (Figure 1d). DRE also reduced the HFD-induced increase in LDL. Both DRE treatments significantly lowered serum LDL levels, and the 1.5% dose showed a slightly better result than the 0.5% dose (Figure 1e).

### 3.2. Effect of DRE on Liver Weight, Hepatic Steatosis, and Histopathological Structure in HED-Fed Mice

**Figure 2:**
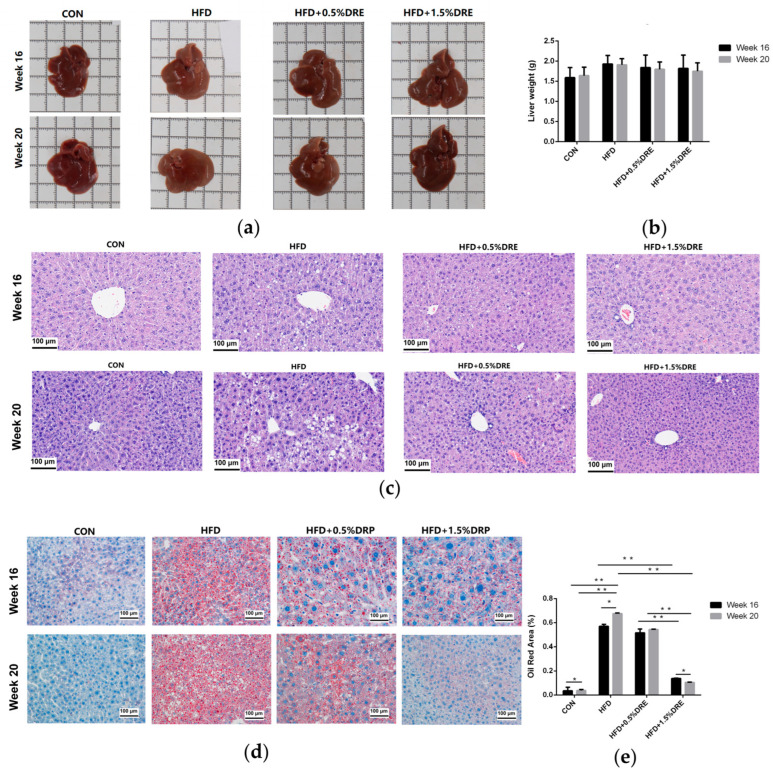
Effects of DRE on hepatic morphology, lipid accumulation, and histological structure in mice. (**a**) Gross morphology of the liver. (**b**) Liver weight. (**c**) H&E staining of liver sections at Week 16 and Week 20. Scale bar = 100 μm. (**d**) Representative images of Oil Red O staining in liver tissues. Scale bar = 100 μm. (**e**) Quantitative analysis of Oil Red O-positive area (%). All data are expressed as the mean ± SD (*n* = 6 per group). * *p* < 0.05 and ** *p* < 0.01.

To assess the protective potential of DRE against HFD-induced liver injury, liver weight, hepatic steatosis and histological alterations were evaluated (Figure 2). Gross measurements indicated that HFD feeding led to an increase in liver weight compared to the CON group (Figure 2a,b). Although DRE intervention resulted in a modest reduction in liver mass, with the 1.5% DRE group exhibiting slightly lower average weights than the HFD group, these values remained elevated relative to the CON group.

Histological examination via Oil Red O staining further revealed the impact of DRE on intrahepatic lipid accumulation. By the end of the 20-week feeding period, livers from the CON group showed sparse red staining with clearly defined hepatocyte structures. In contrast, the HFD group exhibited massive accumulation of red lipid droplets and significant macrovesicular steatosis, characterized by enlarged lipid vacuole size (Figure 2d). Quantitative analysis confirmed that the Oil Red O-positive area in the HFD group was significantly higher than that in the CON group (Figure 2e).

Following 8 weeks of dietary intervention, the 1.5% DRE group showed a marked improvement in hepatic steatosis, evidenced by a significant reduction in the size and density of lipid droplets compared to the HFD group. This therapeutic effect was notably superior to that observed in the 0.5% DRE group. Specifically, while the 1.5% DRE dose significantly lowered the lipid-positive area, the 0.5% DRE group failed to demonstrate a substantial improvement, with the positive area even showing a slight increase at week 20 (Figure 2e). Furthermore, H&E staining corroborated these findings; 1.5% DRE effectively mitigated the disruption of hepatic cords and cellular swelling induced by the HFD (Figure 2c). Collectively, these data suggested that DRE exerted a dose-dependent protective effect against HFD-induced lipid deposition and histological damage in the liver.

### 3.3. Effect of DRE on Fat Accumulation in Mice

**Figure 3:**
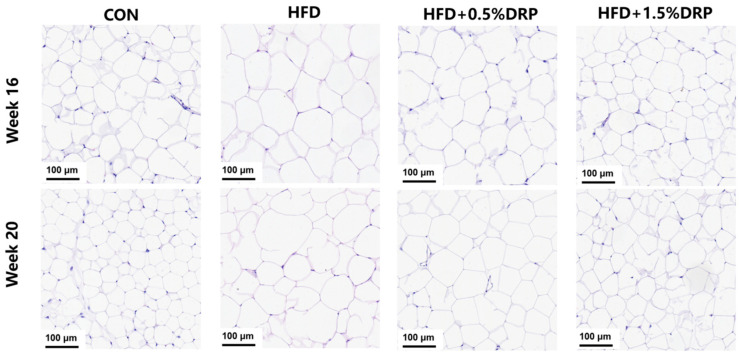
Effect of DRE on the morphology of inguinal white adipose tissue (iWAT) in mice from the CON, HFD, HFD + 0.5% DRE, and HFD + 1.5% DRE groups. Scale bar = 100 μm. Images are representative of six mice per group.

H&E staining of adipose tissue sections was performed to evaluate the impact of DRE on lipid storage and cellular architecture (Figure 3). In the CON group, the adipose tissue structure remained distinct, with adipocytes appearing small, uniform in size, and arranged in an orderly fashion without observable lesions. Conversely, prolonged HFD feeding resulted in a dramatic expansion of adipocyte volume. The cells in the HFD group exhibited pronounced hypertrophy and a crowded arrangement, indicating significant pathological changes associated with excessive lipid accumulation.

Following the dietary intervention, DRE effectively mitigated these morphological alterations. Compared with the HFD group, adipocytes in the 1.5% DRE-treated group appeared smaller and more regularly arranged, with a histological appearance closer to that of the CON group. The reduction in adipocyte size was less prominent in the 0.5% DRE group. These results suggest that DRE supplementation may inhibit fat accumulation and attenuate adipose tissue degeneration.

### 3.4. Effect of DRE on the Gut Microbial Diversity in Mice

To investigate whether the mitigation of HFD-induced obesity by DRE is associated with structural modulations of the gut microbiota, 16S rRNA gene sequencing was performed on fecal samples from the CON, HFD, and HFD + 1.5% DRE groups.

**Figure 4:**
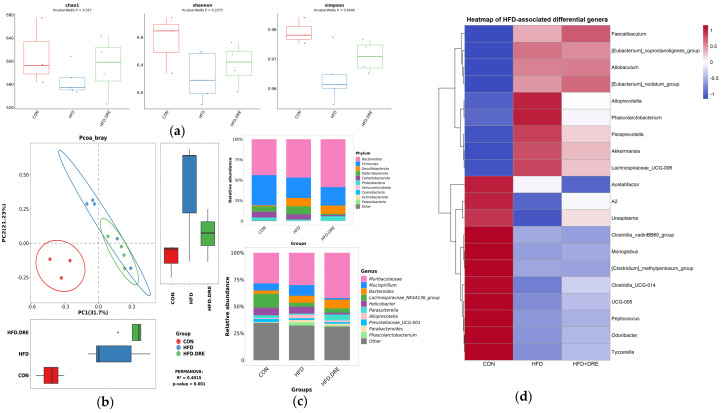
Effects of DRE on gut microbiota in mice (sequencing replicates: *n* = 3 for CON, *n* = 5 for HFD, and *n* = 4 for HFD + 1.5% DRE, derived from 2-by-2 pooled fecal samples). (**a**) Alpha-diversity analysis including Shannon, Simpson, and Chao1 indices. (**b**) Principal Coordinate Analysis (PCoA) illustrating the separation of CON, HFD, and HFD + 1.5% DRE microbial communities. (**c**) Relative abundance of gut microbiota at the phylum level. (**d**) Heatmap of the main genus showing the structural shifts in microbial composition. The color scale represents Z-score-normalized relative abundance for each genus across samples (red, higher relative abundance; blue, lower relative abundance).

Alpha diversity was evaluated using the Chao1, Shannon, and Simpson indices (Figure 4a). Although high-fat diet (HFD) feeding led to a decreasing trend in these indices, which was partially reversed by DRE supplementation, the changes did not reach statistical significance among the groups (Kruskal–Wallis *p* > 0.05).

In contrast, beta diversity analysis using Principal Coordinate Analysis (PCoA) based on Bray-Curtis distances revealed profound shifts in the overall microbial community structure (Figure 4b). The PERMANOVA test indicated a highly significant separation among the three groups (R^2^ = 0.4015, *p* = 0.001). The HFD group clustered distinctly away from the control (CON) group. Notably, while the HFD + DRE group remained separated from the CON group, it exhibited a discernible structural shift away from the HFD group, suggesting that DRE supplementation significantly reshaped the HFD-disrupted gut microbial architecture.

**Figure 5:**
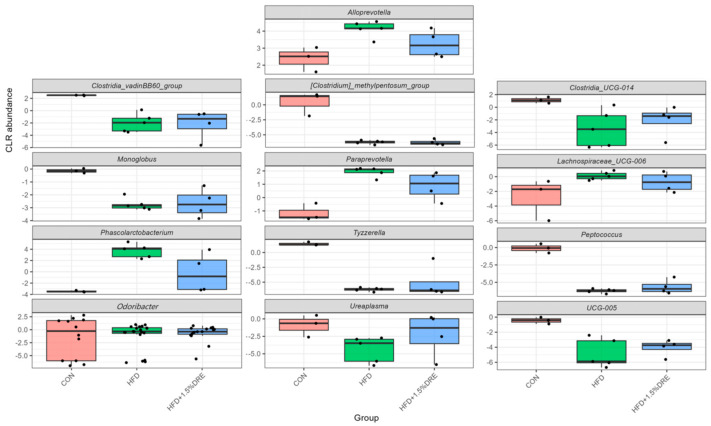
Comparative analysis of fecal microbiota composition in mice. Individual panels illustrate the specific taxonomic shifts identified via ANCOM-BC2 analysis among the CON, HFD, and HFD + 1.5% DRE groups. Box plots represent the median (horizontal line) and the first and third quartiles (box limits), with whiskers extending to 1.5 times the interquartile range. Individual data points are superimposed as overlaid jitter points.

The taxonomic composition was further analyzed at both the phylum and genus levels to map the microbial landscape (Figure 4c). To pinpoint the specific bacterial taxa driving these ecological shifts, a heatmap of HFD-associated differential genera was constructed (Figure 4d), followed by a quantitative validation of their centered log-ratio (CLR) abundances (Figure 5).

The integrated analysis of the heatmap and CLR abundance profiles revealed a striking dual-directional regulatory effect of DRE. On one hand, the HFD intervention provoked an aberrant expansion of obesity- and inflammation-associated taxa. As explicitly demonstrated in the CLR boxplots (Figure 5), taxa such as *Alloprevotella*, *Phascolarctobacterium*, *Paraprevotella*, and *Lachnospiraceae_UCG-006* experienced a massive surge in abundance under HFD feeding. Strikingly, DRE intervention strongly suppressed the HFD-induced overgrowth of these genera, forcing their abundances to significantly regress toward the healthy baseline (CON) levels.

On the other hand, HFD feeding induced a severe depletion of several key endogenous commensals, compromising the gut barrier and metabolic homeostasis. Supplementation with DRE effectively rescued the populations of these beneficial taxa. The quantitative analysis in Figure 5 clearly illustrates this restorative effect, highlighting a robust rebound in the CLR abundances of *Ureaplasma*, *Monoglobus*, and key members of the *Clostridia* class—notably the *Clostridia_UCG-014*, *Clostridia_vadinBB60_group*, and *UCG-005*. Since many of these rescued *Clostridia* taxa are well-documented degraders of complex carbohydrates and producers of short-chain fatty acids (SCFAs), their restoration provides a compelling microbial mechanism for the metabolic improvements observed in the host. Collectively, these highly consistent multi-omics visualizations confirm that DRE intervention mitigates HFD-induced dysbiosis by concurrently inhibiting opportunistic pathogens and revitalizing depleted beneficial commensal networks.

### 3.5. Effect of DRE on Serum Antioxidant Capacity and Inflammatory Status in Overweight Cats

**Figure 6:**
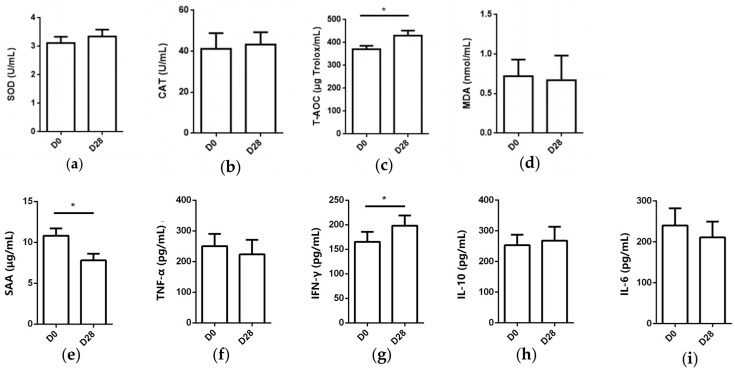
Effects of DRE on serum antioxidant capacity and inflammatory status in overweight cats. (**a**) Superoxide dismutase (SOD) activity. (**b**) Catalase (CAT) activity. (**c**) Total antioxidant capacity (T-AOC). (**d**) Malondialdehyde (MDA) concentration. (**e**) Serum amyloid A (SAA) concentration. (**f**) Tumor necrosis factor-alpha (TNF-α) concentration. (**g**) Interferon-gamma (IFN-γ) concentration. (**h**) Interleukin-10 (IL-10) concentration. (**i**) Interleukin-6 (IL-6) concentration. All data are expressed as the mean ± SD (*n* = 6). * *p* < 0.05 versus Day 0; NS indicates no significant difference (*p* > 0.05).

Serum samples were evaluated to determine the impact of DRE on the systemic antioxidant defense and inflammatory levels (Figure 6).

DRE supplementation induced significant changes in feline serum cytokine profiles. Serum total antioxidant capacity (T-AOC) increased by 16.1% (*p* < 0.05), and the systemic inflammatory marker serum amyloid A (SAA) decreased by 27.8% (*p* < 0.05), indicating reduced systemic inflammation. Concurrently, serum IFN-γ levels increased by 20% (*p* < 0.05), while TNF-α and IL-6 showed downward trends [specify *p*-values], and IL-10 levels remained stable (*p* > 0.05).

Notably, the 28-day DRE intervention maintained the overall physiological stability of the feline subjects, as no significant alterations were observed in body weight, body condition score (BCS), or fecal score throughout the study (*p* > 0.05, Supplementary Table S2). This crucial finding indicates that the observed enhancements in systemic antioxidant capacity and the attenuation of inflammation were direct metabolic benefits of DRE, independent of body weight changes.

### 3.6. Effect of DRE on the Gut Microbial Diversity in Overweight Cats

To evaluate the modulatory effect of DRE on the intestinal microecology of overweight cats, 16S rRNA gene sequencing was performed on fecal samples collected before (D0) and after a 28-day intervention (D28).

**Figure 7:**
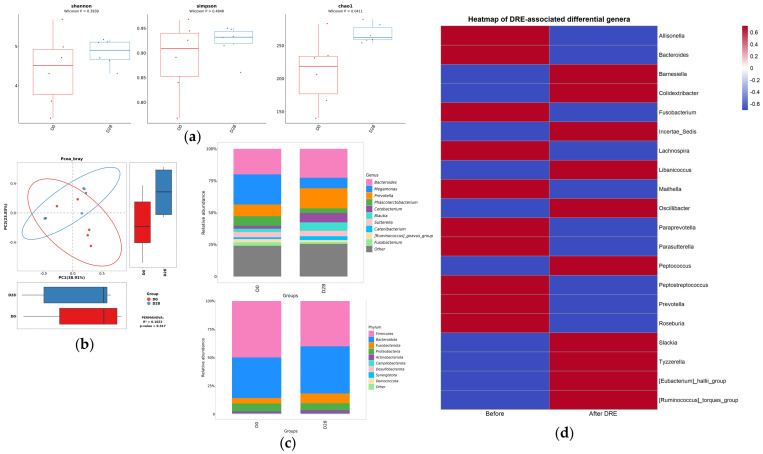
Effects of DRE on gut microbiota in overweight cats (individual samples: *n* = 6 cats at each timepoint, D0 and D28, analyzed without sample pooling or compositing). (**a**) Alpha-diversity analysis including Shannon, Simpson, and Chao1 indices. (**b**) Principal Coordinate Analysis (PCoA) illustrating the separation of D0 and D28 microbial communities. (**c**) Relative abundance of gut microbiota at the phylum level. (**d**) Heatmap of the main genus showing the structural shifts in microbial composition.; taxa with significantly altered abundance between D0 and D28 (Wilcoxon signed-rank test, *p* < 0.05) are highlighted. The color scale represents Z-score-normalized relative abundance for each genus across samples (red, higher relative abundance; blue, lower relative abundance).

Alpha-diversity indices were calculated to characterize microbial richness and evenness (Figure 7a). Following the intervention, the Chao1 index exhibited a statistically significant elevation (Wilcoxon test, *p* = 0.0411), demonstrating that DRE effectively enriched the overall microbial species richness in overweight cats. While the Shannon and Simpson indices displayed numerical upward trends, these changes did not reach statistical significance (*p* = 0.3939 and *p* = 0.4848, respectively). These findings indicate that DRE primarily enhances feline gut microbial diversity by promoting species richness rather than drastically altering species evenness.

To assess the global macroscopic shifts in the microbial community structure, Beta-diversity was evaluated using Principal Coordinate Analysis (PCoA) based on Bray–Curtis dissimilarities (Figure 7b). A PERMANOVA test indicated that the community architecture did not undergo a drastic global shift between Day 0 and Day 28 (R^2^ = 0.1022, *p* = 0.317). This suggests that rather than causing disruptive structural remodeling, the DRE intervention maintained the fundamental ecological stability of the feline gut while selectively modulating specific microbial taxa.

Taxonomic profiling at the phylum level revealed that the feline gut microbiota was predominantly composed of *Firmicutes* and *Bacteroidota* across both time points (Figure 7c). Following the 28-day DRE supplementation, a noticeable remodeling of these dominant phyla was observed, characterized by an expansion in the relative abundance of *Bacteroidota* and a concurrent reduction in *Firmicutes.* This shift resulted in a substantial decrease in the *Firmicutes/Bacteroidota* (F/B) ratio, a widely recognized microbiological hallmark frequently associated with the mitigation of the obesity phenotype. Additionally, a moderate expansion in the relative abundance of Fusobacteriota was observed in the D28 group compared to the baseline.

The heatmap of differential genera (Figure 7d) revealed a targeted and precise modulation of the microbial composition. Paralleling our findings in the mouse model, DRE intervention induced a distinct ecological shift toward a healthier microbial profile. Specifically, the heatmap demonstrated a robust enrichment of several beneficial taxa post-intervention, most notably the short-chain fatty acid (SCFA) producers such as *Oscillibacter*, and *[Eubacterium]_hallii_group (Anaerobutyricum hallii)*. It should be noted, however, that fecal SCFA concentrations were not directly quantified in this study; the proposed SCFA-mediated mechanism is therefore inferred from genus-level taxonomic enrichment of recognized SCFA-producing taxa and is supported by prior literature linking these taxa to SCFA production, but remains to be directly confirmed through targeted metabolomic analysis. Future studies combining 16S sequencing with fecal metabolomics and mechanistic assays (e.g., intestinal barrier function, regulatory T cell differentiation) will be necessary to fully elucidate the role of SCFA in mediating the beneficial effects of DRE. Concurrently, DRE supplementation effectively suppressed the overgrowth of potentially opportunistic pathogens, exemplified by the clear reduction in *Fusobacterium*, and *Bacteroides* compared to the baseline. These results confirm that DRE exerts a conserved regulatory mechanism across species—specifically, by fostering beneficial commensal networks and curbing pro-inflammatory taxa—thereby validating its potential as a safe and effective functional ingredient for optimizing the gut microenvironment in companion animals.

## 4. Discussion

Obesity arises from a chronic energy imbalance where caloric intake consistently exceeds expenditure, leading to systemic metabolic dysfunction and excessive lipid deposition [3,48,49,50]. In this study, the high-fat diet (HFD) group in mice successfully established a robust obesity model, demonstrated by a 10.1% increase in body weight compared to the control group within 12 weeks. Dietary intervention with DRE dose-dependently mitigated further weight gain. While the 0.5% dose had a moderate impact, the 1.5% DRE group showed a more significant reduction, maintaining an average weight 6.2% lower than the HFD group by the end of the 20-week trial.

The liver and adipose tissues are the primary sites for lipid processing, and their structural integrity is a critical indicator of metabolic health [48,51]. In HFD-fed mice, severe hepatic steatosis was confirmed by massive macrovesicular lipid accumulation, with the Oil Red O-positive area reaching 67.5%. Intervention with 1.5% DRE dramatically improved this pathological state, reducing the lipid-positive area to 10.5% and effectively mitigating cellular swelling and the disruption of hepatic cords. Simultaneously, 1.5% DRE inhibited the expansion of adipocytes in white adipose tissue [52]. Adipocytes in the 1.5% DRE-treated group were significantly smaller and more regularly arranged than those in the HFD group, nearly resembling the physiological state of the control group. At the terminal timepoint (week 20), 1.5% DRE attenuated HFD-induced adipocyte hypertrophy compared to the HFD control, demonstrating a dose-dependent protective effect of DRE supplementation on adipose tissue structure [53]. Furthermore, systemic metabolic health was supported by the serum lipid analysis in mice, where 1.5% DRE significantly reduced TC and LDL levels compared to the HFD baseline. These data indicate that DRE exerts a comprehensive protective effect against the structural and metabolic disturbances induced by high-fat intake.

Oxidative stress and chronic low-grade inflammation are hallmark features of obesity [6,52]. In the feline model, DRE intervention significantly increased T-AOC by 16.1%, while SOD and CAT showed non-significant upward trends and MDA marginally declined, suggesting a strengthened antioxidant defense primarily via non-enzymatic mechanisms. Concurrently, systemic inflammation was markedly alleviated, as evidenced by a 27.8% reduction in SAA and downward trends in IL-6 and TNF-α. Serum IFN-γ increased by 20%, an observation that warrants cautious interpretation given its pro-inflammatory context. Importantly, these changes occurred without significant weight loss, underscoring that the metabolic and immune benefits of DRE are independent of weight changes. This antioxidant improvement may help protect the intestinal environment from obesity-associated oxidative stress [54,55].

The gut microbiota is a critical regulator of host energy homeostasis, and its diversity is frequently compromised in obese individuals [56,57]. In the present study, both HFD-induced obese mice and overweight cats exhibited a distinct decline in microbial richness and altered community composition. However, DRE supplementation consistently reversed this dysbiosis. In mice, although alpha-diversity metrics did not show statistically significant changes, beta-diversity analysis revealed a significant taxonomic shift induced by DRE. This ecological remodeling was characterized by the enrichment of depleted beneficial commensals, including *Odoribacter*, *Monoglobus*, and members of the *Clostridia* class, while concurrently suppressing the HFD-induced overgrowth of obesity-associated taxa such as *Alloprevotella* and *Phascolarctobacterium* [58,59]. As primary degraders of complex polysaccharides, these rescued taxa (especially *Odoribacter* and *Clostridia* members) ferment dietary fibers into SCFAs [60], playing vital roles in maintaining intestinal barrier integrity and modulating host lipid metabolism [26,61,62,63,64].

These findings were further corroborated in the feline model. DRE intervention in overweight cats significantly increased the Chao1 index, suggesting that the extract effectively promoted the recovery of species richness. Furthermore, the relative abundance of the *Bacteroidota* phylum increased, a microbial shift commonly associated with lean phenotypes. Taxonomic profiling revealed that DRE enriched specific beneficial taxa, notably the genus *Oscillibacter*. As described in the cited literature, *Oscillibacter* and its related genus *Oscillospira* are robust biomarkers inversely correlated with body weight and lower body mass index (BMI), both genera belong to the family Oscillospiraceae, members of which—along with *[Eubacterium]_hallii_group (Anaerobutyricum hallii)*—are widely recognized as vital contributors to the short-chain fatty acid (SCFA) pool in the mammalian gut [65,66,67]. Heatmap analysis also showed robust enrichment of potent SCFA producers such as *[Eubacterium]_hallii_group*, accompanied by a targeted reduction in *Bacteroides* and *Fusobacterium*. Members of *Oscillospiraceae*, *[Eubacterium]_hallii_group* is widely recognized as vital contributors to the SCFA pool in the mammalian gut [68,69]. Specifically, taxa within *Oscillospiraceae* are potent producers of butyrate [70]. Beyond SCFA production, previous metagenomic studies suggested that *Oscillospiraceae* members can degrade host glycans, which requires the host to expend additional energy to regenerate intestinal mucins [71,72]. This increased energy expenditure provided a potential metabolic mechanism for the weight reduction observed in the feline and murine models. Meanwhile, taxa such as *[Eubacterium]_hallii_group (Anaerobutyricum hallii)* is highly efficient at producing short-chain fatty acids (particularly butyrate) via microbial cross-feeding networks [73].

These enriched SCFAs, particularly butyrate, sustain host health by strengthening the intestinal mucosal barrier and suppressing systemic inflammation [67,74]. In contrast, DRE reduced the abundance of opportunistic pathogens and pro-inflammatory taxa such as *Fusobacterium* in the feline model, as well as *Paraprevotella* and *Lachnospiraceae_UCG-006* in the murine model, which cause gut dysbiosis and mucosal inflammation. By reducing the burden of these pro-inflammatory taxa and promoting the expansion of SCFA-producers, DRE improved the overall gut microbial environment [75,76]. These consistent microbial changes were in alignment with the decreased SAA and increased T-AOC reported earlier, suggesting a strong mechanistic link between gut microbiota remodeling and systemic health improvement during weight management.

## 5. Limitations of the Study

This study has several important limitations that should be acknowledged. First, the feline cohort was small (*n* = 6) and employed a before-and-after self-controlled design without a parallel placebo-fed comparison group. This design limitation should be considered when interpreting the feline microbiota, cytokine, and antioxidant findings, particularly for low-abundance taxa, and may affect the generalizability of the results. Future studies should employ larger, randomized, placebo-controlled, parallel-group designs to strengthen causal inference. Second, the duration of the feline intervention (28 days) was relatively short for assessing durable metabolic effects; longer-term studies with post-intervention follow-up are needed to confirm the durability of the observed antioxidant and anti-inflammatory benefits and to evaluate effects on body weight and adiposity over time. Third, fecal short-chain fatty acid (SCFA) concentrations were not directly quantified in either the murine or feline studies. Although the pronounced enrichment of SCFA-producing taxa *(Oscillibacter*, *[Eubacterium] hallii group*, and *Clostridia* members) suggests a SCFA-mediated mechanism linking microbiota shifts to host metabolic and anti-inflammatory benefits, this mechanism is inferred from taxonomic composition and supporting literature rather than directly measured. Future studies should incorporate targeted fecal metabolomics to quantify major SCFA species (acetate, propionate, butyrate) alongside 16S sequencing to establish mechanistic links between microbiota composition and host phenotype. Fourth, comprehensive chemical characterization of the DRE extract was based on untargeted metabolomics only; targeted quantification of individual bioactive compounds (e.g., deinoxanthin concentration, polyphenol profiles, SCFA precursors) was not performed. Future studies should include high-performance liquid chromatography (HPLC) or liquid chromatography–mass spectrometry (LC-MS) analysis to precisely identify and quantify the active constituents of DRE and enable structure–function correlation with the observed metabolic and immune effects. Finally, this study examined only rodent and companion animal models; the applicability of these findings to human populations remains unclear. Clinical trials in overweight or obese humans will be necessary to evaluate the safety, tolerability, and efficacy of DRE supplementation for translational therapeutic development.

## 6. Conclusions

In conclusion, the present study demonstrates that *Deinococcus radiodurans* extract (DRE) serves as a potent natural intervention for mitigating high-fat diet (HFD)-induced metabolic disorders and optimizing systemic physiological health. In the murine model, 1.5% DRE supplementation successfully counteracted HFD-induced obesity, demonstrated by significantly reduced weight gain, regulated serum lipid profiles, and markedly alleviated pathological lipid deposition in both the liver and adipose tissues. Mechanistically, DRE altered the murine gut microbial community by effectively revitalizing core beneficial commensals depleted by the HFD—notably *Odoribacter*, *Monoglobus*, and various members of the *Clostridia* class—while concurrently suppressing the aberrant overgrowth of obesity- and inflammation-associated taxa such as *Alloprevotella* and *Phascolarctobacterium*.

These health-promoting benefits were successfully translated and validated in the feline model. In naturally overweight domestic cats maintained under stable energy intake, a 28-day DRE intervention significantly enhanced systemic antioxidant capacity (T-AOC) and suppressed chronic low-grade inflammation (SAA). At the microecological level, DRE intervention in felines significantly promoted fecal microbial species richness (Chao1 index) and enriched specific beneficial short-chain fatty acid (SCFA) producers, including *Oscillibacter*, and the *[Eubacterium]_hallii_group (Anaerobutyricum hallii)*, while profoundly restricting the abundance of the pro-inflammatory pathogen Fusobacterium.

Importantly, a cross-species comparison of these microbiome profiles reveals a striking ecological similarity: despite host-specific baseline variations, DRE consistently exerts a highly conserved regulatory mechanism across both mammalian models. This shared microbial signature is universally characterized by the selective expansion of functional, carbohydrate-degrading, and SCFA-producing bacterial networks alongside the parallel suppression of detrimental or opportunistic pathogens. Collectively, these findings highlight the robust efficacy and translational potential of DRE as a safe, novel postbiotic ingredient for weight management, antioxidant defense enhancement, and metabolic health restoration in both laboratory settings and companion animal nutrition.

## Data Availability

The datasets presented in this study can be found in online repositories. The names of the repository/repositories and accession number(s) can be found below: National Microbiology Data Center (NMDC), NMDC40125118–NMDC40125141.

## Supplementary Materials

The following supporting information can be downloaded at: https://www.mdpi.com/article/10.3390/ani16132072/s1, Table S1: Baseline demographics, energy requirements, and dietary intake of the feline subjects; Table S2: Longitudinal physiological and clinical tracking of overweight cats during the 28-day DRE intervention; Table S3: Ingredient composition and energy profile of the experimental diets (g/kg as-fed basis); Table S4: Chemical Composition of Deinococcus radiodurans Extract (DRE) by Non-Targeted Metabolomics.

## Abbreviations

The following abbreviations are used in this manuscript:

ANCOM-BC2	Analysis of Compositions of Microbiomes with Bias Correction 2
DR	*Deinococcus radiodurans*
DRE	*Deinococcus radiodurans* Extract
F/B	*Firmicutes/Bacteroidota* ratio
H&E	Hematoxylin and Eosin
HFD	High-Fat Diet
IL-6	Interleukin-6
IL-10	Interleukin-10
IFN-γ	Interferon-gamma
LDL	Low-density lipoprotein
LDL-C	Low-Density Lipoprotein Cholesterol
MCS	Muscle condition scores
MDA	Malondialdehyde
PCoA	Principal Coordinate Analysis
PERMANOVA	Permutational Multivariate Analysis of Variance
SAA	Serum Amyloid A
SCFA	Short-Chain Fatty Acid
TC	Total Cholesterol
TG	Triglyceride
TNF-α	Tumor Necrosis Factor α
T-AOC	Total Antioxidant Capacity
WAT	White Adipose Tissue
